# Routine Preoperative Oesophagogastroduodenoscopy Before Roux‐En‐Y Gastric Bypass: A Retrospective Cohort Analysis From Queensland, Australia

**DOI:** 10.1111/ans.70927

**Published:** 2026-08-24

**Authors:** Shahab Valizadehzare, Michael Hatzifotis

**Affiliations:** ^1^ Faculty of Medicine University of Queensland Brisbane Queensland Australia; ^2^ Department of Surgery Greenslopes Private Hospital Greenslopes Queensland Australia; ^3^ Department of Surgery Princess Alexandra Hospital Woolloongabba Queensland Australia

**Keywords:** bariatric surgery, endoscopy, gastric adenocarcinoma, intestinal metaplasia, RYGB

## Abstract

**Introduction:**

The role of routine preoperative oesophagogastroduodenoscopy (POOGD) with routine biopsies prior to Roux‐en‐Y gastric bypass (RYGB) remains controversial. Whether POOGD should be routinely offered or reserved for symptomatic and/or at‐risk patients is heavily debated. This study aimed to determine the proportion of patients undergoing RYGB in whom POOGD altered surgical management.

**Methods:**

A retrospective review of medical records from 2016 to 2024 was performed using data from Surgery Brisbane, Queensland, Australia. Demographic data, clinical characteristics, POOGD findings, and operative outcomes were collected. POOGD findings were categorised as benign (e.g., oesophagitis, gastritis, duodenitis, peptic ulcer disease [PUD], hiatus hernia [HH]) or premalignant (e.g., intestinal metaplasia [IM], Barrett's oesophagus [BO] and *Helicobacter pylori* infection [HP]).

**Results:**

A total of 423 patients were planned to undergo RYGB; 346 (81.8%) were female. The majority of patients had three or more comorbidities (81.8%) and a preoperative BMI of ≥ 35 kg/m^2^ (88.2%). POOGD identified abnormalities in 338 patients (80.0%), including benign findings in 235 (55.6%) and premalignant findings in 103 (24.3%). The most common benign findings were oesophagitis (184, 43.5%) and gastritis (175, 41.4%). IM was identified in 54 patients (12.8%), including three (0.7%) with corpus involvement. Amongst patients with premalignant findings, 32 (31.1%) were asymptomatic. POOGD findings altered surgical management in 18 patients (4.2%): 14 (3.3%) underwent RYGB plus resection of the remnant stomach (RRS), two (0.5%) experienced a delay due to untreated PUD, and two (0.5%) were offered an alternative bariatric procedure. Four hundred and five patients (95.7%) proceeded with RYGB as planned.

**Conclusion:**

RYGB alters gastric anatomy, rendering the remnant stomach inaccessible postoperatively. Preoperative detection of premalignant lesions may be uncommon but clinically valuable, particularly given that such findings can be asymptomatic and may represent precursors to gastric adenocarcinoma. Considering its safety, diagnostic value, and potential to alter surgical management, the findings of this study support the routine use of POOGD with routine biopsies prior to RYGB.

## Graphical abstract

Impact of routine POOGD with routine biopsies on surgical management in 423 patients undergoing RYGB.

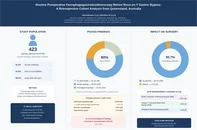

## 1 Introduction

The role of preoperative oesophagogastroduodenoscopy (POOGD) prior to bariatric surgery remains controversial, particularly regarding whether it should be routinely performed in all patients or selectively offered only to those with symptoms or recognised risk factors [1, 2, 3, 4, 5, 6, 7, 8, 9, 10, 11]. This debate is further complicated by the fact that patients may harbour premalignant gastric pathology without symptoms prior to carcinogenesis [8].

POOGD enables detection of clinically relevant upper gastrointestinal pathology, including benign inflammatory conditions (e.g., oesophagitis and gastritis), anatomical abnormalities (e.g., hiatus hernia), premalignant lesions such as intestinal metaplasia (IM), as well as other conditions that lead to an increased risk of developing gastric adenocarcinoma (GAC) such as *Helicobacter pylori* (HP) infection [1, 2, 3, 12, 13]. Studies have shown that approximately 11% of the general population can harbour IM, a recognised precursor to GAC, the incidence of which is increasing globally [12, 13]. British Society of Gastroenterology guidelines [12] recommend POOGD as the standard tool for IM detection, highlighting that IM involving the body of the stomach confers a higher malignant risk than IM in the antrum or the incisura of the stomach as well as calling for HP eradication.

Several studies and professional societies, including national institute of health (NIH) 1991 guidelines, The Obesity Society (TOS), American Society for Metabolic and Bariatric Surgery (ASMBS), International Federation of Surgery for Obesity and Metabolic Disorders (IFSO), Obesity Medicine Association (OMA), and American Society for Gastrointestinal Endoscopy (ASGE) recommend POOGD prior to bariatric operations, recognising that findings may alter surgical planning or uncover pathologies requiring attention before or during the bariatric procedure [2, 5, 10, 11, 14].

While there are many studies advocating for a selective approach [4]—reserving POOGD for patients who are symptomatic or have specific risk factors such as male sex, dyspepsia, NSAID use, or a family history of gastric adenocarcinoma (GAC)—these risk factors are common or near‐universal in the bariatric population, limiting their discriminatory value [7].

Furthermore, large systematic reviews demonstrate that although most endoscopic findings do not affect surgical planning, a clinically meaningful minority do, with rare but significant implications for cancer prevention and long‐term safety [3, 8, 9].

In this context, this retrospective single‐practice audit aims to evaluate the clinical significance and impact of routine POOGD findings on surgical management in patients undergoing RYGB. The authors of this study believe that routine POOGD with routine biopsies, in accordance with the updated Sydney protocol [15], can be a beneficial preoperative step to RYGB, as it has the potential to unmask significant premalignant findings. Hence, the primary outcome of this study was to determine the proportion of RYGB candidates in whom POOGD altered their operation.

## 2 Methods

### 2.1 Study Setting, Participants

During this retrospective study, data extraction was performed by the study investigators using a standardised data collection framework. These de‐identified datasets were then collated and used for statistical analysis and interpretation of the predefined outcome measures. Given the retrospective design, data were limited to what was documented in the medical record.

The dataset comprised medical records of 423 patients who met eligibility criteria for RYGB based NIH 1991 guidelines, American Society of Metabolic and Bariatric Surgery (ASMBS), and International Federation of Surgery for Obesity and Metabolic Disorders (IFSO) guidelines [16, 17] and underwent RYGB at Surgery Brisbane Private Practice (Dr Michael Hatzifotis), located at Greenslopes Private Hospital, Brisbane, Queensland, Australia, between 2016 and 2024.

The extracted variables included demographic data (age, sex, body mass index [BMI], and American Society of Anesthesiologists Physical Status Classification [ASA]), clinical characteristics (obesity‐associated comorbidities and prior bariatric surgical history), preoperative oesophagogastroduodenoscopy (POOGD) findings, and operative outcomes. ASA scores were determined by an anaesthetic doctor either pre‐ or intra‐operatively.

Comorbidities, including gastro‐oesophageal reflux disease (GORD), were identified from medical records based on documented clinical diagnoses and/or associated symptoms recorded by a qualified clinician, using the Modified DeMeester questionnaire. Prior surgical history was obtained from medical records, including previous laparoscopic sleeve gastrectomy (LSG) or laparoscopic adjustable gastric banding (LAGB), where Roux‐en‐Y gastric bypass (RYGB) was considered for revisional surgery.

POOGD findings included both macroscopic and microscopic abnormalities, comprising benign conditions (oesophagitis, gastritis, duodenitis, peptic ulcer disease (PUD), and hiatus hernia (HH)) and premalignant lesions (intestinal metaplasia (IM), *Helicobacter pylori* (HP) and Barrett's oesophagus (BO)). All patients were offered POOGD with routine biopsies, in accordance with the updated Sydney protocol [15], unless OGD had been performed within 18 months prior to surgery without evidence of premalignant pathology.

Histopathological findings were reported by multiple pathologists across different centres without standardisation. Inflammatory conditions (oesophagitis, gastritis and duodenitis) were defined based on macroscopic and/or microscopic findings.

The gastro‐oesophageal junction (GOJ) was assessed in accordance with British Society of Gastroenterology [18] and American College of Gastroenterology [19] guidelines. Targeted biopsies were obtained from columnar‐lined mucosa proximal to the GOJ suspicious for BO. IM at the gastric cardia was carefully distinguished from IM within the tubular oesophagus to avoid misclassification.

HH was identified based on endoscopic visualisation of proximal displacement of the GOJ relative to the diaphragmatic hiatus, as documented in endoscopy reports. However, HH was only repaired when hiatus laxity was laparoscopically evident.

In patients where HP was detected on POOGD, treatment was initiated in accordance with therapeutic guidelines. Given the interval between preoperative assessment (i.e., including POOGD) and the surgery, this did not necessitate alteration of the planned RYGB. These patients were subsequently retested, via urea breath test (UBT), to confirm successful eradication.

In patients with IM, findings were interpreted in accordance with British Society of Gastroenterology guidelines on gastric adenocarcinoma (GAC) [12], with patients stratified into low‐ and high‐risk groups based on anatomical distribution and associated risk factors (e.g., past or family history of GAC). Patients with high‐risk IM (i.e., IM in the body of the stomach, or IM in the antrum/incisura with additional risk factors) underwent RYGB with resection of the remnant stomach (RRS). Patients with low‐risk IM were offered a shared decision regarding proceeding with standard RYGB versus RYGB with RRS, and some elected to undergo RRS to minimise potential future risk. In selected low‐risk IM cases, alternative bariatric procedures (e.g., LSG) were also discussed as part of shared decision making. No cases of dysplastic IM were identified; all IM detected on POOGD was non‐dysplastic.

Among patients with premalignant lesions, medical records were reviewed for upper gastrointestinal (UGI) symptoms, defined as reflux, regurgitation, dysphagia, and post‐prandial epigastric pain, as documented during routine preoperative assessment.

In patients with PUD located in regions of the stomach not typically resected during RYGB, surgery was delayed and repeat endoscopy was performed to confirm ulcer healing and/or regression prior to proceeding. However, in some patients, PUD was identified in the body of the stomach in conjunction with IM. In these cases, surgical planning was not altered, as these segments would have been removed during resectional RYGB regardless.

Operative outcomes included the status of the planned RYGB procedure (performed as planned, altered, delayed, or cancelled), as recorded in the operative reports.

### 2.2 Outcome Measures

The primary measure of this study was to determine the prevalence of premalignant findings identified on POOGD that had the potential to alter the planned RYGB surgery.

Secondary outcome measures included the prevalence of specific POOGD findings, including oesophagitis, gastritis, duodenitis, peptic ulcer disease, hiatus hernia, Barrett's oesophagus, intestinal metaplasia, *Helicobacter pylori* infection and malignancy.

Additionally, the impact of POOGD findings on surgical planning was assessed. This was defined by changes in the status of the planned RYGB procedure, including whether the surgery was performed as planned, altered, delayed, or cancelled based on endoscopic findings.

### 2.3 Analysis

Descriptive statistics were used. Data were analysed using Microsoft Excel for Microsoft 365 MSO (ver. 2405 Build 16.0.17628.20006), 64‐bit. The study was designed to describe prevalence and clinical impact, not test associations or predictors, hence, inferential statistical testing was not performed.

### 2.4 Ethics

Ethical approval was granted by the Ramsay Health Care Human Research Ethics Committee A (HREC reference number: 2025/ETH/0070) under the Low or Negligible Risk review pathway.

## 3 Results

### 3.1 Patient Demographics

Patient demographics are shown in Table 1. Of the 423 patients who were planned to undergo RYGB between 2016 and 2024, 346 (81.8%) were female and 77 (18.2%) were male. As shown in Table 1, except for patients aged 60 years and above (26, 6.1%), increasing age was positively associated with an increasing number of patients in each decade of life.

**TABLE 1:** Demographic data of patients who underwent Roux‐en‐Y gastric bypass (RYGB) surgery at Surgery Brisbane Clinic between 2016 and 2024.

Variables	Cases *N* (%)
Overall	423 (100)
Gender	
Female	346 (81.8)
Male	77 (18.2)
Age	
< 30	53 (12.5)
30–39	86 (20.3)
40–49	127 (30.0)
50–59	131 (31.0)
> 60	26 (6.1)
BMI^a^ (kg/m^2^)	
20–24.9	2 (0.5)
25–29.9	9 (2.1)
30–34.9	39 (9.2)
35–39.9	88 (20.8)
40–49.9	189 (44.7)
> 50	96 (22.7)
ASA^b^	
1–2	224 (53.0)
3+	199 (47.0)

*Note:* Cases are presented as *N*, the number of cases and (%), the percentage.

^a^ Body mass index kg/m^2^ (BMI).

^b^ American Society of Anaesthesiologists Physical Status classification (ASA PS Classifications) simply referred to as ASA.

Preoperative BMI is illustrated in Table 1. Overall, 373 patients (88.2%) had a preoperative BMI ≥ 35 kg/m^2^, 39 patients (9.2%) had BMI between 30 and 34.9 kg/m^2^, and 11 patients (2.6%) had BMI of 20–29.9 kg/m^2^ (Table 1).

As shown in Table 1, patients classified as healthy or having mild systemic disease (ASA 1–2) slightly outnumbered those with severe systemic disease (ASA 3+; 224, 53.0% vs. 199, 47.0%).

### 3.2 Clinical Characteristics

A total of 346 patients (81.8%) had three or more comorbidities (Table 2). As illustrated in Table 2, GORD was the most commonly recorded comorbidity, affecting 275 patients (65.0%), followed by mental health conditions (anxiety and depression; 267, 63.1%) and hypertension (HTN; 176, 41.6%).

**TABLE 2:** Clinical characteristics of Roux‐en‐Y gastric bypass (RYGB) candidates.

Variables	Cases *N* (%)
Comorbidities^a^	
0–2	77 (18.2)
3+	346 (81.8)
GORD^b^	275 (65.0)
OSA^c^	157 (37.1)
T2DM^d^	97 (22.9)
IGT^e^	31 (7.3)
HTN^f^	176 (41.6)
HF^g^	5 (1.2)
FLD^h^	143 (33.8)
PCOS^i^	52 (12.3)
MH^j^	267 (63.1)

Past bariatric surgery history	Cases *N* (%)
Patients with previous bariatric surgery	165 (39.0)
LSG^k^	99 (23.4)
LAGB^l^	66 (15.6)
Patients with no previous bariatric surgery	258 (61.0)

*Note:* Cases are presented as *N*, the number of cases and (%), the percentage.

^a^ Comorbidities refer to obesity‐associated conditions such as.

^b^ Gastroesophageal reflux disease (GORD).

^c^ Obstructive sleep apnea (OSA).

^d^ Type 2 diabetes mellitus (T2DM).

^e^ Impaired glucose tolerance (IGT).

^f^ Hypertension (HTN).

^g^ Heart failure (HF).

^h^ Fatty liver disease (FLD).

^i^ Polycystic ovarian syndrome (PCOS).

^j^ Mental health (MH) conditions (depression and anxiety only).

^k^ Laparoscopic sleeve gastrectomy (LSG).

^l^ Laparoscopic adjustable gastric banding (LAGB).

In terms of metabolic disease, 97 patients (22.9%) had a formal diagnosis of Type 2 diabetes mellitus (T2DM), while an additional 31 patients (7.3%) had impaired glucose tolerance (IGT) (Table 2). Furthermore, 52 patients (12.3%) had polycystic ovarian syndrome (PCOS).

While 258 patients (61.0%) had not undergone any previous bariatric surgery, 165 patients (39.0%) had a history of bariatric procedures, including 99 patients (23.4%) who underwent laparoscopic sleeve gastrectomy (LSG) and 66 patients (15.6%) who underwent laparoscopic adjustable gastric banding (LAGB) (Table 2).

### 3.3 POOGD Results

Table 3 and Figure 1 present POOGD findings. Eighty‐five patients (20.1%) had no abnormalities detected. The remaining 338 patients (80.0%) had one or more benign and/or premalignant findings detected through POOGD. Benign findings were reported in 235 patients (55.6%), while premalignant findings were identified in 103 patients (24.3%).

**TABLE 3:** Preoperative oesophagogastroduodenoscopy (POOGD) findings.

Patients with	Cases *N* (%)
NAD^a^	85 (20.1)
Benign and/or premalignant findings	338 (80.0)
Benign	235 (55.6)
Oesophagitis	184 (43.5)
Gastritis	175 (41.4)
Duodenitis	15 (3.5)
PUD^b^	6 (1.4)
HH^c^	139 (32.9)
Premalignant	103 (24.3)
HP^d^	20 (4.7)
BO^e^	29 (6.9)
Long	7 (30.4)
Short	13 (60.9)
Ultra‐short	2 (8.7)
IM^f^	54 (12.8)
Low risk	
In the antrum or incisura^g^	47 (11.1) [87.0]
High risk	
In the antrum or incisura + risk factors^h^	4 (0.9) [7.4]
In the body^i^	3 (0.7) [5.6]
IM + HP	4 (7.4)

*Note:* Cases are presented as *N*, the number of cases and (%), the percentage.

^a^ No abnormalities detected (NAD).

^b^ Peptic ulcer disease (PUD).

^c^ Hiatus hernia (HH).

^d^ *H. pylori* (HP).

^e^ Barrett‘s oesophagus (BO) was further classified into long (> 3 cm), short (1–3 cm), and ultra‐short segments (< 1 cm), note that only 23 out of 29 BO cases had information regarding the length hence the percentages are calculated out of 23.

^f^ Intestinal metaplasia (IM), note that [%] indicates the percentage subtypes of IM (i.e., IM in the antrum or incisura; IM in the antrum or incisura + risk factors, or IM in the body of the stomach) within the total number of patients with IM (54). IM was further divided into two categories: low risk (Antrum or Incisura of the stomach) and high risk (antrum or incisura + risk factors or IM in the body).

^g^ Antrum or Incisura of the stomach.

^h^ Risk factors that made the patient more prone to gastric malignancy such as a family history of gastric cancer, other red flags (e.g., hematochezia), or previous treated IM.

^i^ The body of the stomach.

**FIGURE 1:**
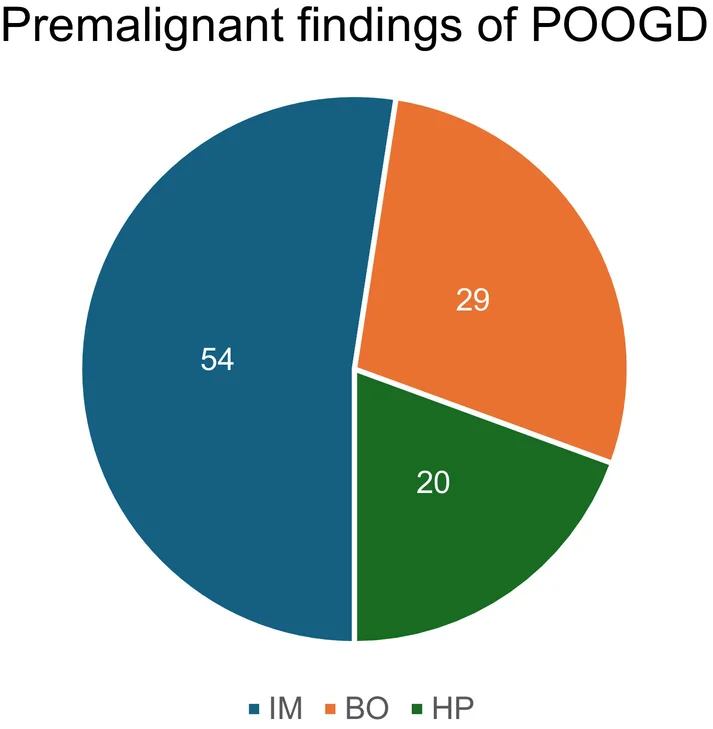
Premalignant findings of preoperative oesophagogastroduodenoscopy (POOGD) such as intestinal metaplasia (IM), Barrett's oesophagus (BO) and *H. pylori* (HP).

Among benign findings, the most commonly detected pathology was oesophagitis (184, 43.5%), followed by gastritis (175, 41.4%). Peptic ulcer disease (PUD) was observed in 6 patients (1.4%). Lastly, hiatus hernia was identified in 139 patients (32.9%).

Furthermore, when focusing on premalignant findings, *Helicobacter pylori* (HP) infection was detected in 20 patients (4.7%), while Barrett's oesophagus (BO) was identified in 29 patients (6.9%). Data regarding the length of BO were available for 23 patients. Long segment BO (> 3 cm) was identified in 7 patients (30.4%), while short segment BO (1–3 cm) was identified in 14 patients (60.9%). Ultra‐short segment BO (< 1 cm) was identified in 2 patients (8.7%) (Table 3).

Intestinal metaplasia (IM) was identified in 54 patients (12.8%). Of these, 47 cases (11.1% of the total cohort and 87.0% of patients with IM) had IM confined to the antrum or incisura, placing them at low risk for the development of GAC. In contrast, four patients (0.9% of the total cohort and 7.4% of patients with IM) had IM in the antrum or incisura in addition to other risk factors (i.e., a first‐degree relative with a history of GAC). These patients, along with three others (0.7% of the total cohort and 5.6% of patients with IM) in whom IM was identified in the body of the stomach, were considered to be at high risk of GAC. Moreover, 4 patients (7.4% of the patients with IM) had IM with concurrent HP infection and colonisation of the stomach (Table 3).

Table 4 demonstrates the demographic composition of patients with IM. IM was identified in 42 female patients (42/346, 12.1%) and 12 male patients (12/77, 15.6%). Patients aged 50–59 years demonstrated the highest prevalence of IM (22/131, 16.8%), whereas patients aged 30–39 years had the lowest prevalence (5/86, 5.8%). Additionally, increasing BMI categories generally demonstrated a higher number of IM cases, except for patients with BMI > 50 kg/m^2^, in whom fewer cases were observed. The highest prevalence of IM was observed in patients with a BMI of 35–39.9 kg/m^2^ (13/88, 14.8%) and 40–49.9 kg/m^2^ (28/189, 14.8%).

**TABLE 4:** Demographic composition of patients with intestinal metaplasia.

Patients with IM^a^	Cases *n*/*N* (%)
Gender	
Female	42/346 (12.1)
Male	12/77 (15.6)
Age	
< 30	5/53 (9.4)
30–39	5/86 (5.8)
40–49	19/127 (15.0)
50–59	22/131 (16.8)
> 60	3/26 (11.5)
BMI^b^ (kg/m^2^)	
20–24.9	0/2 (0.0)
25–29.9	0/9 (0.0)
30–34.9	4/39 (10.3)
35–39.9	13/88 (14.8)
40–49.9	28/189 (14.8)
> 50	9/96 (9.4)

*Note:* Cases are presented as *n*, the number of cases, *N*, the total number of cases in the cohort and (%), the percentage within that group.

^a^ Intestinal metaplasia (IM).

^b^ Body mass index kg/m^2^ (BMI).

As demonstrated by Table 5, among the 103 patients with premalignant POOGD findings, 71 (68.9%) reported UGI symptoms. In contrast, 32 patients (31.1%) with premalignant findings were asymptomatic.

**TABLE 5:** Symptomatic versus non‐symptomatic patients with premalignant findings such as *H. pylori* , Barrett's oesophagus, or intestinal metaplasia, on preoperative oesophagogastroduodenoscopy (POOGD).

Symptom status	Cases (*n* | %)
Total	103 (100)
Symptomatic	71 (68.9)
Asymptomatic	32 (31.1)

### 3.4 The Effect of POOGD on RYGB

Table 6 and Figure 2 summarise the impact of POOGD findings on surgical management. The majority of patients (405, 95.7%) proceeded with RYGB as originally planned while 18 (4.2%) were altered. Fourteen patients (3.3%) underwent resection of the remnant stomach (RRS) in addition to RYGB based on POOGD findings. Of these, seven patients (1.7% of the total cohort) required RRS because IM was identified in the antrum or incisura of the stomach. Four patients (0.9%) underwent RRS due to additional risk factors in the presence of IM affecting the antrum or incisura, while three patients (0.7%) underwent RRS due to IM located in the body of the stomach.

**TABLE 6:** Impact of preoperative oesophagogastroduodenoscopy (POOGD) findings on surgical management in patients planned to undergo Roux‐en‐Y Gastric Bypass (RYGB).

Outcome	Cases (*n* | %)
Operations carried out as planned	405 (95.7)
Operations altered	18 (4.2)
RRS^a^	14 (3.3)
IM^b^ in the antrum or incisura^c^ (low risk)	7 (1.7)
IM in the antrum or incisura + risk factors^d^(high risk)	4 (0.9)
IM in the body^e^(high risk)	3 (0.7)
Delayed (PUD^f^ surveillance)	2 (0.5)
Changed to LSG^g^ (IM in the antrum or incisura)	2 (0.5)

^a^ Resection of the remnant stomach (RRS).

^b^ Intestinal metaplasia (IM).

^c^ Antrum or incisura of the stomach.

^d^ Risk factors that made the patient more prone to gastric malignancy such as a family history of gastric cancer, other red flags (e.g., hematochezia), or previous treated IM.

^e^ The body of the stomach.

^f^ Peptic ulcer disease (PUD).

^g^ Laparoscopic sleeve gastrectomy (LSG).

**FIGURE 2:**
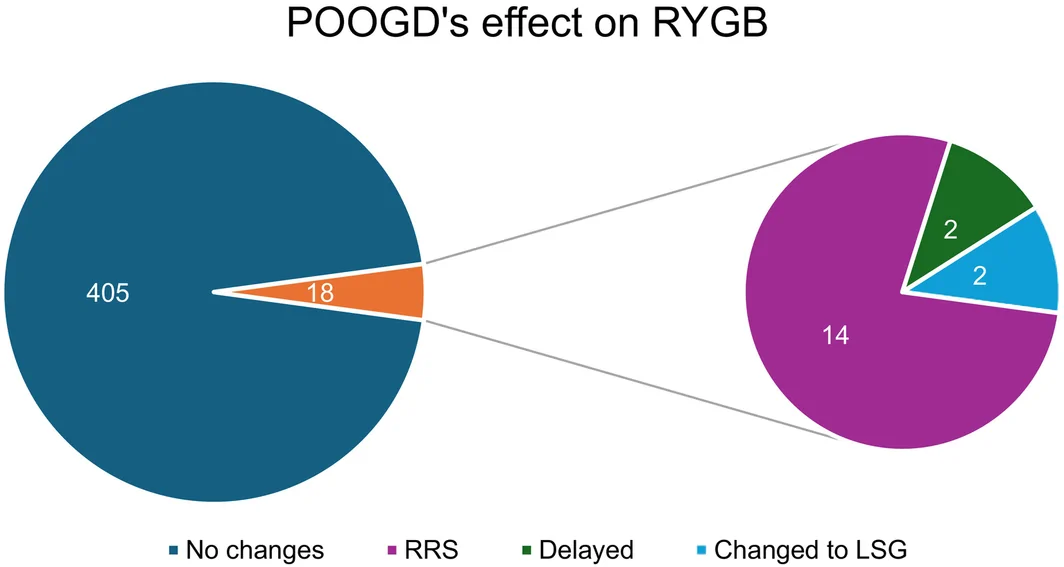
The effect of preoperative oesophagogastroduodenoscopy (POOGD) on the outcome of Roux‐en‐Y Gastric Bypass (RYGB); Resection of the remnant stomach (RRS), Laparoscopic sleeve gastrectomy (LSG). The blue section of the pie chart on the left demonstrates the surgeries that were performed as planned. The orange section of this pie chart however, displays the total proportion of cases in which the surgery was modified. This is then further broken down to the pie on the right. The pie on the right illustrates the different changes including RYGB +RRS (purple), delayed RYGB (green), or RYGB operation changed to a LSG operation (blue).

Two patients (0.5%) experienced a delay in surgery due to untreated PUD identified in POOGD. An additional two patients (0.5%) were offered an alternative bariatric procedure (laparoscopic sleeve gastrectomy, LSG) following identification of IM located in the antrum or incisura of the stomach.

## 4 Discussion

This study hypothesised that preoperative oesophagogastroduodenoscopy (POOGD) prior to Roux‐en‐Y gastric bypass (RYGB) is an important investigation, as it has the potential to identify occult premalignant lesions such as intestinal metaplasia (IM), or *Helicobacter pylori* (HP) infection, all of which may contribute to gastric adenocarcinoma (GAC) carcinogenesis [12].

Elevated BMI is an independent risk factor for GORD [20, 21, 22]. Given that many RYGB candidates have elevated BMI, the high prevalence of oesophagitis observed in this study (43.5%) was not an unexpected finding.

Gastritis is among the most frequently reported gastropathies on OGD [3, 10, 14]. However, the prevalence of HP infection in this study was considerably lower (4.7%). This suggests that the observed gastritis may more strongly reflect obesity‐related pathophysiology rather than HP infection alone [20, 23, 24, 25].

Hiatus hernia (HH), another condition strongly associated with obesity, was identified in 32.9% of patients, consistent with previous studies [3, 10]. However, endoscopic identification of HH does not always correlate with clinical symptoms or intraoperative findings at the gastro‐oesophageal junction (GOJ). In this cohort, HH repair was performed only when hiatus laxity was confirmed laparoscopically. Therefore, HH repair was not classified as an alteration of preoperative surgical planning, as the decision to repair was made intra‐operatively rather than being directly guided by POOGD findings. Hence, the primary aim of this study was to assess changes in surgical management resulting from intragastric pathology detected on POOGD, particularly pathology associated with an increased risk of GAC (e.g., IM). Consequently, addition of HH repair to RYGB was not considered a modification of surgical planning and was not included in the results of this study.

HP infection is a well‐established risk factor for GAC [26]. RYGB surgery renders the remnant stomach inaccessible for postoperative surveillance of HP [3, 12, 27]. Additionally, untreated HP infection has been associated with increased postoperative complications, including bleeding, ulceration and anastomotic leak [10, 27]. Hence, detection and eradication of HP represent a key justification for routine POOGD with routine biopsies, in accordance with the updated Sydney protocol [15], prior to RYGB.

In this cohort, POOGD was performed following British Society of Gastroenterology guidelines [18] and American College of Gastroenterology [19], with additional biopsies from columnar‐lined mucosa at the tubular oesophagus proximal to the GOJ, suspicious of BO. According to both the British Society of Gastroenterology and American College of Gastroenterology guidelines [18, 19], long segment Barrett's oesophagus (BO) is defined as BO extending ≥ 3 cm, whereas short segment BO refers to segments < 3 cm in length. However, there is a lack of consensus on whether ultra‐short segments < 1 cm should be diagnosed as BO [18, 19, 28]. Regardless, BO represents another obesity‐associated condition that increases the risk of oesophageal adenocarcinoma [3, 12, 20, 29]. Chronic upper gastrointestinal (UGI) symptoms such as reflux and regurgitation—typically associated with GORD—can lead to inflammatory changes of the oesophagus, including the development of BO [28]. However, due to its anti‐reflux properties, RYGB has been shown to significantly reduce UGI symptoms (e.g., reflux, regurgitation, dysphagia) and has also been associated with regression of BO, supporting its potential as a therapeutic option in this population [28, 30].

Peptic ulcer disease (PUD), associated with both HP infection and obesity‐related gastritis [20, 27], poses a risk for postoperative complications and gastric cancer, particularly when located in the remnant stomach [3, 27, 31]. Ulcers in these locations may necessitate a delay in surgery to allow for ulcer healing prior to definitive intervention.

There is limited evidence to support the diagnosis of intestinal metaplasia (IM)—an advanced premalignant lesion and precursor to gastric adenocarcinoma (GAC)—without POOGD and histological assessment [12]. The updated Sydney protocol [15], widely used by pathologists for reporting gastric biopsies, mandates documentation of IM presence and location. Given its premalignant nature, identification of IM necessitates modification of surgical planning. British Society of Gastroenterology guidelines recommend that RYGB be adjusted based on its location [12, 13]. IM confined to the antrum or incisura without additional risk factors does not necessitate alteration of RYGB or postoperative surveillance [12, 13, 32]. However, IM at these sites in the presence of risk factors (e.g., family history of gastric cancer) warrants either postoperative surveillance or resection of the remnant stomach (RRS) at the time of RYGB [12, 13]. IM involving the gastric body is an absolute indication for RRS regardless of additional risk factors [12, 13]. The prevalence of IM in this cohort is consistent with prior studies [33] and reinforces the importance of routine histological assessment, particularly given its premalignant potential. Older age and male sex have been associated with both IM and GAC [12, 34, 35]. Although this study was limited to descriptive analysis, in which no associations can be inferred, the highest prevalence of IM was found amongst patients aged 50–59 years (16.8%), while the lowest prevalence was observed amongst patients aged 30–39 years (5.8%). Proposed explanations for these differences include genetic factors [35]; however, environmental and lifestyle factors may also contribute. In particular, cigarette smoking, which has historically been more prevalent among males [36], has been identified as a risk factor for both IM and GAC [37].

One of the most debated aspects of POOGD prior to bariatric surgery is whether its use should be guided by symptoms [3, 5, 11, 13, 33, 38]. The proportion of symptomatic and asymptomatic patients with premalignant conditions in this cohort is consistent with previous studies [11, 12, 15, 20, 39], which demonstrate that gastritis, HP infection, and IM are often asymptomatic or minimally symptomatic. These findings underscore the importance of routine POOGD prior to RYGB, as the procedure may render premalignant lesions permanently inaccessible regardless of symptomatology.

Of course, it is important to acknowledge that POOGD carries its own risks and disadvantages. Although uncommon (< 2%), complications related to sedation (e.g., cardiovascular events such as hypoxia, arrhythmia, or airway obstruction), the procedure itself (e.g., bleeding and/or perforation), and the post‐procedural period (e.g., infection) may occur [40, 41]. In addition, POOGD may delay the primary operation (i.e., RYGB), thereby increasing waiting times and potentially disrupting the flow of care. Finally, POOGD represents a significant additional cost and burden to both the patient and the healthcare system.

Lastly, it is important to acknowledge the limitations of this study. First, this audit was conducted within a single surgical practice in Queensland, Australia, which limits the generalisability of the findings to other public and private healthcare settings. Second, the retrospective design exposes the results to potential documentation errors and reporting bias and precludes the establishment of causality. In addition, the retrospective nature of the study limited long‐term follow‐up, preventing assessment of the progression of IM to GAC. Furthermore, as this was not a randomised clinical trial, there was no control group with which to compare outcomes or healthcare costs.

Nevertheless, although POOGD altered surgical management in only 4.2% of cases, this represents 18 patients in whom POOGD findings directly influenced operative management. Importantly, even minor changes in preoperative assessments may translate into tangible reductions in clinical risk in a cohort undergoing irreversible anatomical alteration.

In conclusion, findings of this study, in accordance with the updated Sydney protocol [15], support the routine use of POOGD with routine biopsies prior to RYGB, irrespective of the presence or absence of UGI symptoms suggestive of benign or premalignant upper gastrointestinal pathology. Routine POOGD with routine biopsies, in accordance with the updated Sydney protocol [15], represents a low‐risk and clinically valuable approach for detecting premalignant lesions that may otherwise remain undiagnosed in the surgically inaccessible remnant stomach, thereby posing a significant risk to patient outcomes in the postoperative period.

## Author Contributions

All authors contributed to the study design, manuscript review, and agree to be accountable for the final manuscript.

## Funding

The authors have nothing to report.

## Disclosure

The authors have nothing to report.

## Conflicts of Interest

The authors declare no conflicts of interest.

## Data Availability

The data that support the findings of this study are available on request from the corresponding author. The data are not publicly available due to privacy or ethical restrictions.
